# Evidence-based clinical practice guidelines for inflammatory bowel disease 2020

**DOI:** 10.1007/s00535-021-01784-1

**Published:** 2021-04-22

**Authors:** Hiroshi Nakase, Motoi Uchino, Shinichiro Shinzaki, Minoru Matsuura, Katsuyoshi Matsuoka, Taku Kobayashi, Masayuki Saruta, Fumihito Hirai, Keisuke Hata, Sakiko Hiraoka, Motohiro Esaki, Ken Sugimoto, Toshimitsu Fuji, Kenji Watanabe, Shiro Nakamura, Nagamu Inoue, Toshiyuki Itoh, Makoto Naganuma, Tadakazu Hisamatsu, Mamoru Watanabe, Hiroto Miwa, Nobuyuki Enomoto, Tooru Shimosegawa, Kazuhiko Koike

**Affiliations:** 1 Guidelines Committee for Creating and Evaluating the “Evidence-Based Clinical Practice Guidelines for Inflammatory Bowel Disease”, The Japanese Society of Gastroenterology, 6F Shimbashi i-MARK Building, 2-6-2 Shimbashi, Minato-ku, Tokyo, 105-0004 Japan; 2 Department of Gastroenterology and Hepatology, Sapporo Medical University School of Medicine, S-1, W-16, Chuoku, Sapporo, Hokkaido 060-8543 Japan

**Keywords:** Inflammatory bowel disease, Steroid, Immunomodulators, Biologics

## Abstract

Inflammatory bowel disease (IBD) is a general term for chronic or remitting/relapsing inflammatory diseases of the intestinal tract and generally refers to ulcerative colitis (UC) and Crohn’s disease (CD). Since 1950, the number of patients with IBD in Japan has been increasing. The etiology of IBD remains unclear; however, recent research data indicate that the pathophysiology of IBD involves abnormalities in disease susceptibility genes, environmental factors and intestinal bacteria. The elucidation of the mechanism of IBD has facilitated therapeutic development. UC and CD display heterogeneity in inflammatory and symptomatic burden between patients and within individuals over time. Optimal management depends on the understanding and tailoring of evidence-based interventions by physicians. In 2020, seventeen IBD experts of the Japanese Society of Gastroenterology revised the previous guidelines for IBD management published in 2016. This English version was produced and modified based on the existing updated guidelines in Japanese. The Clinical Questions (CQs) of the previous guidelines were completely revised and categorized as follows: Background Questions (BQs), CQs, and Future Research Questions (FRQs). The guideline was composed of a total of 69 questions: 39 BQs, 15 CQs, and 15 FRQs. The overall quality of the evidence for each CQ was determined by assessing it with reference to the Grading of Recommendations Assessment, Development and Evaluation approach, and the strength of the recommendation was determined by the Delphi consensus process. Comprehensive up-to-date guidance for on-site physicians is provided regarding indications for proceeding with the diagnosis and treatment.

## Introduction

- Purpose of the revised guidelines
  The purpose of these practice guidelines is to improve patient outcomes by providing appropriate practice measures for health care providers and patients for the treatment of inflammatory bowel disease (IBD).
- Basic policy
  In accordance with the policy of the previous guideline, the basic concepts of the Grading of Recommendations Assessment, Development and Evaluation (GRADE) system, which has been used in many foreign guidelines, were incorporated as much as possible to create a medical index that emphasizes the totality of evidence from systematic reviews[1].
- Development method
  In the actual preparation of the guideline, we held a series of face-to-face preparation committee meetings and e-mail deliberations, prepared draft questions, and formulated items. The Clinical Questions (CQs) of the previous guideline were completely revised and categorized as follows:
- Background questions (BQs): those for which conclusions are already clear, and those for which a consensus has already been reached in previous guidelines.
- CQs: questions that affect the direction of medical treatment and for which recommendations and evidence levels can be determined by an exhaustive literature search.
- Future research questions (FRQs): questions for which a recommendation and level of evidence cannot be determined by the current exhaustive literature search (no evidence exists.)

This guideline includes 69 questions: 39 BQs, 15 CQs, and 15 FRQs. A literature search was created for each question, and for CQs and FRQs, the search period was from 1983 to April 2019 for English articles and from 1983 to May 2019 for Japanese articles. The Japan Association of Medical Libraries was commissioned to conduct a literature search in PubMed and the Central Journal of Medicine. For the CQs, three meta-analyses were prepared and have just published. For BQs, references were manually searched by each committee member, and no search period was applied. The statement and commentary were completed. The overall quality of the evidence for each CQ was determined by assessing it with reference to the GRADE approach (Table 1). The strength of the recommendation was determined by the the drafting committee using the Delphi method (Table 2). The appropriateness of the wording of the statement was independently evaluated by 17 members of the drafting committee. A rating scale of 9 (9 = most appropriate, 1 = most inappropriate) was used, with a median rating of 9 or 8 indicating a strong recommendation and a median rating of 7 indicating a weak recommendation. As a result, a consensus recommendation (median value of 7 or higher) was obtained for all statements, but there were variations in the ratings for some statements, requiring reevaluation to reach a consensus.

**Table 1:** Quality of evidence

A: High quality evidence	We are confident that the true effect approximates the effect estimates
B. Moderate quality evidence	Moderate confidence in the effect estimates. The true effect is approximately close to the effect estimate, but it may be substantially different
C. Low quality evidence	Confidence in the estimated effect is limited The true effect may be substantially different from the effect estimate
D. Very-low-quality evidence	Effect estimates are largely unreliable The true effect is likely to be substantially different from the effect estimate

**Table 2:** Strength of recommendation

Grade of recommendation	Criteria (mean Delphi score)	Interpretation
1. Strong recommendation	8–9	Recommendation to do Recommendation not to do
2. Weak recommendation	7	Suggest to do Suggest not to do

The draft was submitted to the evaluation committee, and after the evaluation comments were collected, feedback was given to the drafting committee members in charge, and necessary revisions were made. This process was repeated once more, and the final draft was developed. The final draft was posted on the website of the Japanese Society of Gastroenterology from August 3 to 17, 2020, for public comment.

4. Application of guidelines

This guideline is intended to support decision-making in clinical practice by describing standard information on the disease concept, diagnosis, treatment, and follow-up of IBD. The Japanese Gastroenterological Association (JGAA) and this Guideline Development and Evaluation Committee are not responsible for the results of individual treatment. The Japanese Society of Gastroenterology and the Committee for the Preparation and Evaluation of this guideline are not responsible for the results of individual treatment. The contents of this guideline are not to be used as a legal basis for medical litigation.

5. Structure of the medical algorithm

In this guideline, the following treatment algorithm is presented in a flowchart (nine figures).

The algorithm is simplified to the maximum extent possible, although treatment may be complicated in IBD, where treatment options vary by disease state.

## Definition and pathophysiology of IBD

**BQ 1 What is IBD?**

### Statements

- IBD is a general term for chronic or remitting/relapsing inflammatory diseases of the intestinal tract and generally refers to ulcerative colitis (UC) and Crohn's disease (CD).
- UC is a diffuse, nonspecific inflammation of unknown origin that continuously damages the colonic mucosa from the rectal side, often leading to erosions and ulcers.
- CD is a chronic inflammatory disease of unknown etiology characterized by noncontiguous distributed, all-stratified granulomatous inflammation and fistulae.

These statements and supplementary were made with reference to [2, 3]

### Supplementary information

Cases of enteritis that cannot be differentiated as UC or CD are referred as follows:

1. IBD unclassified (IBDU): this term is used for patients who do not have a surgical specimen available (i.e., have not undergone surgery) and whose diagnosis is difficult to make despite a combination of clinical, endoscopic and histological findings.
2. Indeterminate colitis: as a rule, a surgical specimen is used for the diagnosis of indeterminate colitis and is used in cases with characteristics of UC and CD.

**BQ 2. What is the epidemiology of IBD in Japan?**

### Statements

- The number of IBD patients is estimated to be more than 220,000 for UC and more than 70,000 for CD, based on the current number of medical certificates issued.
- Both UC and CD occur at a relatively young age, with a high incidence in the late teens to early 30 s.

These statements were made with reference to [4, 5]

**BQ 3. What are the factors that contribute to and exacerbate IBD?**

### Statements

- Multiple loci have been reported to be associated with the development of UC and CD.
- Although the cause of UC/CD is unknown, an association with certain dietary factors has been reported.
- Smoking and appendicectomy have been reported to be protective against UC.
- Current smoking has been reported to be a risk factor for the development of CD.
- Oral contraceptives and nonsteroidal anti-inflammatory drugs (NSAIDs) have been reported to be associated with the development of IBD.
- The pathogenesis of IBD is associated with dysbiosis.

These statements were made with reference to the following information and paper [6–15]

## Diagnosis

**BQ 4. How do you proceed with the diagnosis of IBD?**

### Statements

- The diagnosis of IBD is established by suspicion based on the information obtained in the medical interview, characteristic findings of IBD in the physical examination and the endoscopic and other imaging study results typical of IBD.
- Persistent or recurrent bloody diarrhea with abdominal pain and frequent bowel movements should lead to the suspicion of IBD, especially in young patients.
- A problem in the differentiation of IBD is infectious enteritis.
- Chronic abdominal pain, diarrhea, bloody stools, weight loss, fever, and anal lesions should lead to the suspicion of IBD, especially in young patients.

**Fig. 1:**
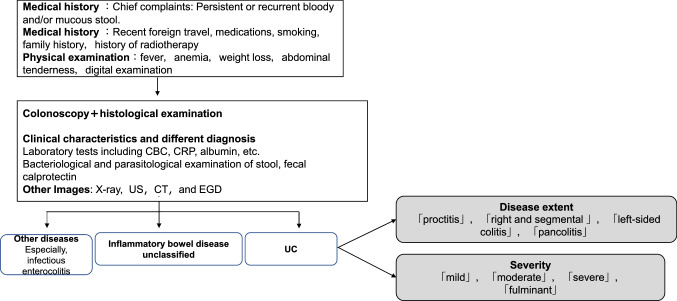
Diagnostic approach for ulcerative colitis. Mild to moderate active left-sided colitis type (not extending beyond the sigmoid colon) and proctitis type

**Fig. 2:**
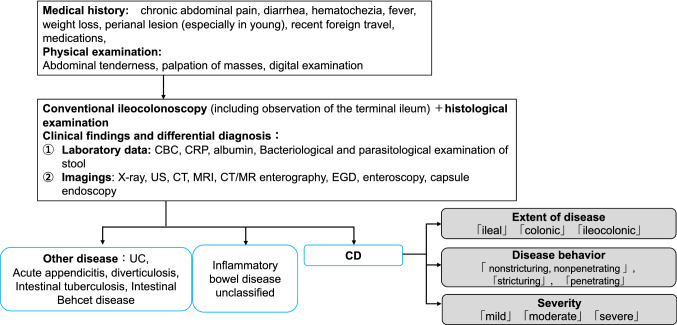
Diagnostic approach to Crohn's disease

These statements were made with reference to [2, 3]. Please refer to Figs. 1 and 2.

**BQ 5. What are the diagnostic criteria for IBD?**

### Statement

- A diagnosis of IBD is made following the diagnostic criteria of the Ministry of Health, Labour, and Welfare's “Research on Intractable Inflammatory Bowel Disorders”.

This statement was made with reference to [16]. Please refer to Tables 3 and 4.

**Table 3:** Diagnostic criteria for ulcerative colitis [16]

Diagnostic criteria for ulcerative colitis
*A. Clinical manifestations: persistent or recurrent mucous or bloody stools, or a history of them*
*B. Laboratory findings* 1. Endoscopic examination a) The mucosa is diffusely affected, angiogenesis has disappeared, and the mucosa is coarse or granular. b) Multiple erosions, ulcers or pseudopolyposis are present. c) Basically, the lesion is continuous with the rectum 2. Ba enema a) Diffuse changes on the mucosal surface in the form of coarse or fine granules and b) multiple erosions, ulcers or pseudopolyps. Other findings include the loss of the haustra (lead tube) and the narrowing and shortening of the intestine
*C. Histopathological findings:* In the active phase, there is a diffuse inflammatory cell infiltration of all layers of the mucosa, crypt abscesses and a high degree of goblet cell depletion. All of these findings are nonspecific and should be judged in the aggregate. In remission, the glands remain misaligned (tortuous or branched) and atrophic. The above changes are usually seen from the rectum to the mouth in a continuous fashion

Confirmed diagnosis of ulcerative colitis

(1) In addition to A, 1 or 2 of B and C are fulfilled

(2) 1 or 2 of B and C on more than one occasion

(3) Patients with gross and histological findings, which are characteristic of the disease on resection or autopsy

**Table 4:** Diagnostic criteria for Crohn’s disease (reference 16)

Diagnostic criteria for Crohn's disease
*Main findings* A. Longitudinal ulcer (in the case of the small intestine, preferably on the mesenteric attachment) B. Cobble stone appearance C. Noncavitary epithelioid cell granuloma: serial sectioning of histology samples improves the diagnostic yield. The diagnosis should be made by a pathologist familiar with the gastrointestinal tract
*Secondary findings* a. Extensive irregular to round ulcers or aphthae in the gastrointestinal tract: extensive gastrointestinal tract lesions means that the lesions are anatomically distributed over more than one organ, i.e., the upper gastrointestinal tract (esophagus, stomach, duodenum), small intestine and large intestine. The lesions are typically longitudinal, but may not be longitudinal. The disease should be permanent for at least 3 months. On capsule endoscopy, there may be multiple rings in the Kerckring folds of the duodenum and small intestine. It is necessary to exclude intestinal tuberculosis, intestinal Behçet's disease, simple ulcer, nonsteroidal anti-inflammatory drug ulcers and infectious enteritis b. Characteristic anorectal lesions: anal fissures, cavitating ulcers, hemorrhoids, perianal abscesses, edematous cortices, etc. We recommend that physicians ask an anorectologist familiar with Crohn's disease and use the Crohn's Disease Atlas of Anorectal Lesions to confirm the diagnosis c. Characteristic gastric and duodenal lesions: bamboo like appearance, notch-like depressions. The diagnosis should be made by a specialist in Crohn's disease

Confirmed diagnosis of Crohn’s disease

1. Patients with major findings A or B. If there is only a longitudinal ulcer, ischemic bowel disease or ulcerative colitis should be excluded. If only a cobblestone appearance is present, ischemic bowel lesions and type 4 colorectal cancer should be excluded

2. The patient must have a primary finding of C and a secondary finding of a or b

3. Patients with all of the secondary findings (a, b, and c)

Note 1: Inflammatory bowel disease unclassified may develop more characteristic features of one of these diseases with follow-up

**BQ 6. What is the pathology, classification, and severity of UC?**

### Statements

- There are two phases of UC: the active phase, in which symptoms are present, and the remission phase, in which symptoms disappear.
- UC can be divided into three types according to the extent of the lesion: “proctitis”, “left-sided colitis” (up to the splenic flexure), and “total colitis”.
- The severity of UC is classified as “mild”, “moderate”, or “severe” based on clinical symptoms, signs, and blood tests (Table 5).
- Depending on the disease's clinical course of UC, the disease is classified as relapsing–remitting, chronically persistent, acutely fulminant, or first attack types.

These statements and supplementary information were made with reference to [16–20]. Please refer to Table 5.

**Table 5:** Classification of severity of ulcerative colitis [16]

	Severe	Moderate	Mild	
(1) Bowel movements	≧ 6		≦ 4	(1) Bowel movements
(2) Blood in stools	(+++)		(+)~(−)	(2) Blood in stools
(3) Pyrexia	≧ 37.5 °C	Between mild and moderate	No	(3) Pyrexia
(4) Pulse	≧ 90/min		No	(4) Pulse
(5) Anemia	Hb ≦ 10 g/dL		No	(5) Anemia
(6) ESR	≧ 30 mm/h		Normal	(6) ESR
or CRP	≧ 3.0 mg/dL		Normal	or CRP

Patients are classified as severe if they present both (1) and (2) plus at least one of (3) or (4)

While satisfying 4 or more out of 6 features, patients with extremely severe symptoms are classified as fulminant, and further divided into acute fulminant or relapsing fulminant types. Diagnostic criteria of fulminant colitis: all of the below:

(1) Satisfy criteria of severe cases

(2) Bloody diarrhea 15 or more times day continuously

(3) Persistent high fever ≧ 38.0 °C

(4) White blood cell count ≧ 10,000/mm^3^

(5) Severe abdominal pain

ESR, erythrocyte sedimentation rate; CRP, C-reactive protein

### Supplementary information

The partial Mayo score is a four-point scale that incorporates the frequency of bowel movements, rectal bleeding, and the physician's general assessment of the patient's condition. A score of 0–1 indicates remission, 2–4 indicates mild disease, 5–7 indicates moderate disease, and 8 or more indicates severe disease (Table 6).

**Table 6:** Partial Mayo score [19]

Mayo items	Clinical assessment
Stool frequency	0 = Normal 1 = 1–2 stools/day more than normal 2 = 3–4 stools/day more than normal 3 = > 4 stools/day more than normal
Rectal bleeding^a^	0 = None 1 = Visible blood with stool less than half the time 2 = Visible blood with stool half of the time or more 3 = Passing blood alone
Physician rating of disease activity	0 = Normal 1 = Mild 2 = Moderate 3 = Severe

^a^A score of 3 for bleeding required patients to have at lease 50% of bowel movements accompanied by visible blood and at least one bowel movement with blood alone

**BQ 7. What is the pathology, classification, and severity of CD?**

### Statements

- The most common sites of CD are in the small and large intestine (especially in the ileum) and perianal region and are classified as “ileal-type”, “colonic -type”, and “ileocolonic- type”.
- It has been proposed to classify the disease pattern of CD in three ways: “non-stricturing, non-penetrating type”, “penetrating type”, and “stricturing type” (Table 7).
- The Crohn's Disease Activity Index (CDAI) (Table 8), the International Organization for the Study of Inflammatory Bowel Disease (IOIBD) index, and the Harvey-Bradshaw index have been proposed as CD activity indicators. Nevertheless, they have not been widely used in general practice.

**Table 7:** Montreal classifications for Crohn’s disease [21]

Clinical factors	
Age at diagnosis	A1: below 16 years A2: between 17 and 40 years A3: above 40 years
Disease location	L1: ileal L2: colonic L3: ileocolonic L4: isolated upper disease
Disease behavior	B1: nonstricturing, nonpenetrating B2: stricturing B3: penetrating ‘p’: perianal disease modifier

L4 is a modifier that can be added to L1-3 when concomitant upper gastrointestinal disease is present. ‘p’ is added to B1-3 when concomitant perianal disease is present

**Table 8:** Classification of severity of Crohn’s disease [16, 23]

	CDAI	Complication	Inflammation	Response to treatment
Mild	150–220	No	Slightly elevated	
Moderate	220–450	No clinically significant complications (e.g., bowel obstruction)	Significantly elevated	Not responding to mild treatments
Severe	450<	Significant complication (e.g., bowel obstruction, abscess formation)	Extremely elevated	Refractory

These statements were made with reference to [16, 21–24]. Please refer to Tables 7 and 8.

## Endoscopy and other imaging modalities

**BQ 8. What is the role of endoscopy in the diagnosis and treatment of UC?**

### Statements

- Colonoscopy is necessary to confirm the diagnosis if UC is suspected based on clinical findings.
- Colonoscopy is used to confirm the diagnosis of UC and to evaluate the severity of the disease, determine the effectiveness of treatment, and conduct surveillance for carcinogenesis.

These statements were made with reference to [2, 16, 19, 25–33].

**BQ 9. What are nonendoscopic, noninvasive tests used in the diagnosis of UC?**

### Statement

- Noninvasive abdominal ultrasound (US), computed tomography (CT) and magnetic resonance imaging (MRI) scans are used to assess activity and confirm complications before and after treatment.

This statement was made with reference to [16, 26, 34–38].

**BQ 10. What is the role of endoscopy in the diagnosis and treatment of CD?**

### Statements

- If CD is suspected, lower gastrointestinal (GI) endoscopy (including observation of the ileum's distal end) and histopathological examination with biopsy should be performed.
- Upper GI endoscopy is recommended, especially if the diagnosis cannot be confirmed by lower GI endoscopy or if the patient complains of upper GI symptoms.
- Endoscopy is performed when necessary to confirm the diagnosis of CD and evaluate the severity of the disease, determine the effectiveness of treatment, and conduct surveillance for carcinogenesis.
- Balloon-assisted enteroscopy or small-bowel capsule endoscopy (SBCE) may be useful for the close examination and follow-up of small bowel lesions in CD.

These statements were made with reference to [16, 25, 39–49].

**CQ 1. Is SBCE useful for the assessment of small bowel disease activity in CD?**

### Recommendation

- SBCE is as useful as CT enterography (CTE) and magnetic resonance enterography (MRE) for the assessment of small bowel disease activity or postoperative recurrence in patients with CD -confirmed bowel patency. **[Strong recommendation, moderate-quality evidence]**

### Commentary

In the meta-analysis comparing the diagnostic yield of active small bowel lesions [50], SBCE was reported to demonstrate a better diagnostic yield when comparing small-bowel follow-through or enteroclysis. The yield was no different compared with that of CTE or MRE. In cross-sectional studies comparing the yields between SBCE and MRE [51, 52], SBCE has been reported to show better diagnostic yields, especially in the upper part of the small bowel [52]. However, the results should be interpreted cautiously, because the types and the severity of small-bowel lesions detected by SBCE are different from those detected by cross-sectional studies. Another meta-analysis demonstrated that SBCE, MRE and US showed favorable diagnostic yields of anastomotic recurrence in patients with CD who underwent ileocecal resection [53]; however, the difference in the definition of postoperative recurrence among the studies could cause selection bias.

The diagnostic yield of SBCE for CD reportedly varies from 20 to 86% in suspected CD [54]. Such a difference might be because the diagnosis of CD cannot be confirmed by SBCE findings alone, and because the definition of small-bowel lesions was different among the studies. SBCE findings that can be useful for the distinction of CD have been recently proposed [49]. It is necessary to determine the usefulness of SBCE for the diagnosis of CD based on certain SBCE findings. Another report concluded that SBCE is not recommended for patients with negative CTE or enteroclysis findings when considering cost-effectiveness [55].

The association of SBCE findings with clinical disease activity and biomarkers has been scarcely investigated. A cross-sectional study reported a positive association [56], while another cohort study failed to show a correlation between the severity of SBCE and of biomarkers (C-reactive protein (CRP), erythrocyte sedimentation rate (ESR), fecal calprotectin). The risk of capsule retention has been reportedly high in both established CD (5–13%) and suspected CD (4–13%), however, the risk can be minimized with the application of a patency capsule (PC) beforehand. While the confirmation of small-bowel patency by using a PC is thus recommended, we should consider the impaction of the PC itself.

**BQ 11. What are imaging studies other than endoscopy are used to diagnose CD?**

### Statements

- Radiographic and other imaging studies are used to determine treatment strategy and to determine the extent, severity, and complications of the lesion.
- US, CT, and MRI are mainly used to evaluate patient disease activity before and after treatment and to check for complications.

These statements were made with reference to [16, 26, 35, 37, 47, 57–64].

**CQ 2. Is MRI useful for the evaluation of CD disease activity?**

### Recommendation

- The use of MRE or magnetic resonance enterocolonography (MREC) is recommended for monitoring intestinal disease activity, evaluating mucosal healing and extraluminal disease, and evaluating treatment response. **[Strong recommendation, moderate-quality evidence]**

### Commentary

MRE and MREC are useful in assessing for small-bowel lesions that are difficult to visualize with endoscopy. MRE and MREC are able to detect lesions in the small and large bowel with a high degree of accuracy, and are useful for diagnosis, monitoring for disease activity, and evaluating treatment response in CD [65]. The presence of edema, wall thickening of more than 3 mm, contrast enhancement, stenosis, and fistula are assessed [66]. A meta-analysis of MRE and MREC studies reported a sensitivity of > 80% and specificity of > 90% for the detection of inflammation and a sensitivity of > 90% and specificity of > 95% for the detection of intestinal damage such as abscesses and fistulae [67].

Several scores have been developed for the assessment of disease activity, the most frequently validated of which is the Magnetic Resonance Index of Activity (MaRIA) [68]. The MaRIA was shown to correlate well with the CD Endoscopic Index of Severity (CDEIS) both before and after treatment in a prospective study, detecting endoscopic mucosal healing with a sensitivity of 85% and specificity of 78% [69]. In addition, the modified MaRIA correlated well with the Simple Endoscopic Score for Crohn’s Disease (SES-CD), and a sensitivity of 87% and specificity of 86% were reported for mucosal healing [70]. In recent years, several simpler scores have been developed, all of which show a high correlation with endoscopy and the MaRIA [71, 72]. The presence of disease activity upon MRE significantly correlates with relapse, including postoperative relapse, and surgery [73, 74]. For disease monitoring, modalities such as MRE/MREC and intestinal US are recommended, especially in cases where repeated examinations are required and in patients under 35 years of age, where radioactive exposure should be minimized [75]. MRE/MREC has been associated with problems of access and training, and a consensus statement has been issued regarding the procedure, imaging sequence and interpretation [76].

## Biomarkers

**BQ 12. Is fecal calprotectin testing useful for the differential diagnosis of IBD?**

### Statement

- Fecal calprotectin testing is useful for differentiating between organic intestinal diseases such as IBD and functional intestinal diseases such as irritable bowel syndrome (IBS).

This statement was made with reference to [65, 77–79].

**BQ13. Are fecal calprotectin tests and fecal immunochemical tests (FITs) useful for assessing disease activity in UC patients in remission?**

### Statement

- Fecal calprotectin tests and FITs (hemoglobin concentrations in feces measured by using an antibody for human hemoglobin) are useful for evaluating the disease activity in UC patients in the remission stage.

This statement was made with reference to [80–84].

## Treatment

**BQ14. What is “Treat to Target” (T2T) in IBD treatment?**

### Statements

- The concept of T2T is when physicians and patients discuss treatment goals and review treatment options at appropriate intervals using a comprehensive activity index to achieve early clinical remission or low disease activity.
- Prospective observational studies are required to determine whether the treatment goals for UC and CD proposed by the Selecting Therapeutic Targets in Inflammatory Bowel Disease (STRIDE) program contribute to improved patient quality of life.

These statements and supplementary information were made with reference to [30, 31, 85–90].

### Supplementary information

The STRIDE program has been implemented at the IOIBD [87]. The aim of this program is to use an evidence-based consensus to identify therapeutic targets that would be useful in the implementation of T2T therapy in clinical practice. The results of the STRIDE program are as follows: (1) T2T in UC is aimed at achieving no rectal bleeding, improving diarrhea, improving defecation habits (decrease in frequency) and improving findings on endoscopy (Mayo score 0–1), with histological remission as an adjunct goal. T2T in CD is aimed at improving abdominal pain and diarrhea, improving defecation habits (decrease in frequency) and improving ulceration findings on ileal and colonoscopy, or improving inflammatory findings on cross-sectional imaging (CT/MRI/US) in patients in whom lesions cannot be assessed by lower endoscopy up to the terminal ileum. Calprotectin levels can serve as adjunctive targets.

## Current treatment strategy of IBD

**Fig. 3:**
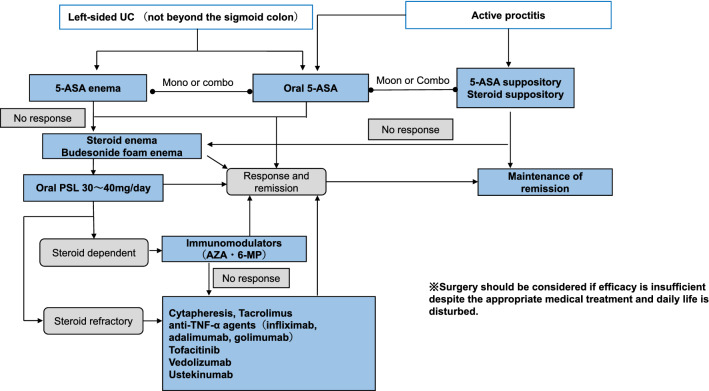
Remission induction therapy for ulcerative colitis

**Fig. 4:**
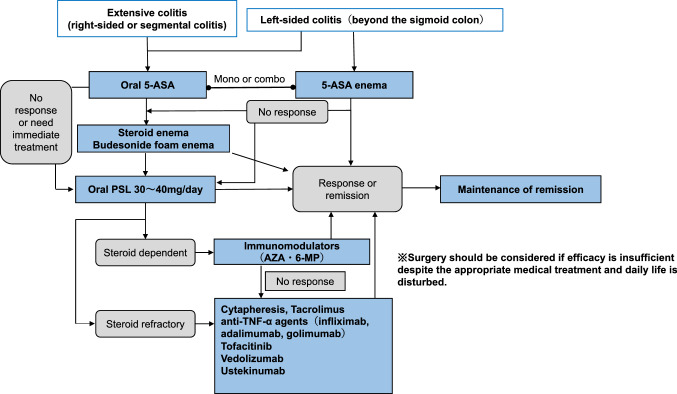
Mild to moderate active-stage total colitis type, right-sided or regional colitis type remission induction therapy for left-sided colitis type (beyond sigmoid colon) ulcerative colitis

**Fig. 5:**
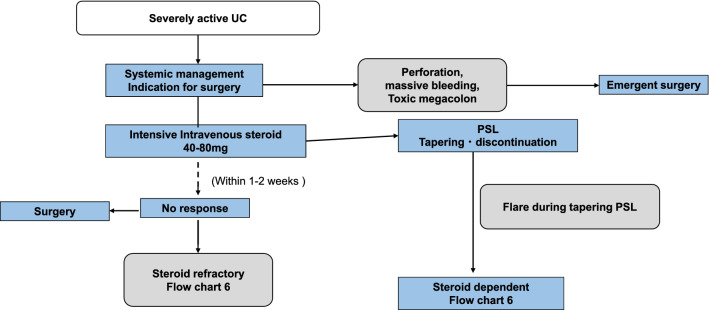
Treatment for severe ulcerative colitis

**Fig. 6:**
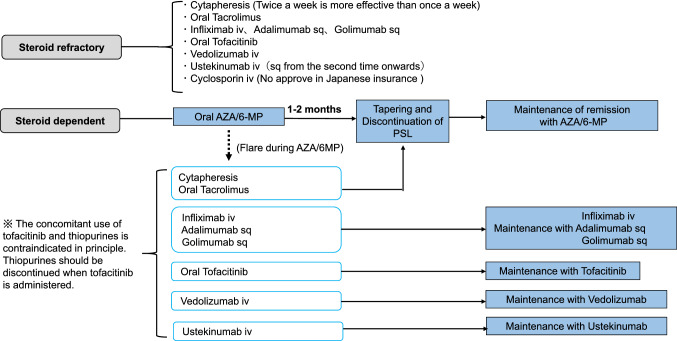
Treatment of refractory cases of ulcerative colitis (including maintenance therapy)

**Fig. 7:**
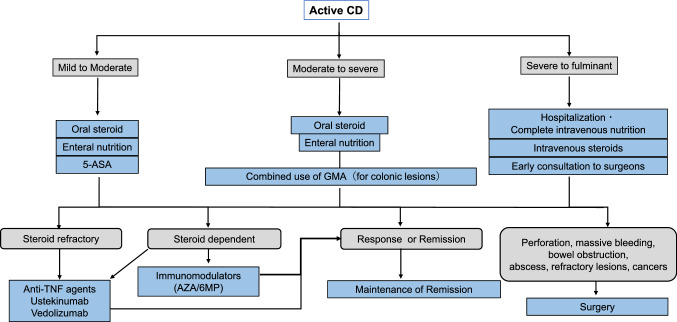
Induction of remission for active Crohn's disease

**Fig. 8:**
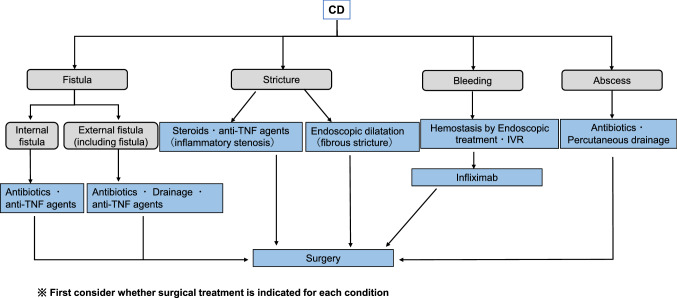
Treatment of gastrointestinal complications of Crohn's disease

**Fig. 9:**
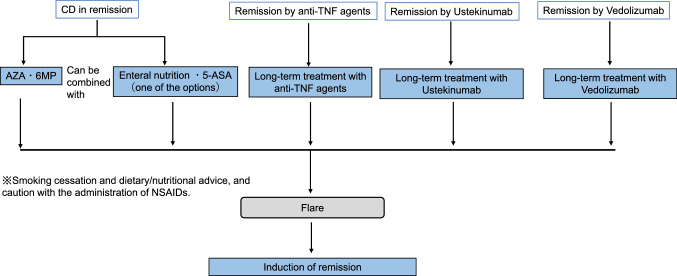
Maintenance therapy for Crohn's disease in remission

- Curative medical therapy has not been established for IBD patients.
- At present, medical treatment goals are early induction of remission and long-term maintenance to prevent relapse.
- In the active stage, it is necessary to accurately diagnose the patient's general condition and the extent of the disease and proceed with treatment based on the treatment guidelines proposed by the Ministry of Health, Labour and Welfare Grant-in-Aid for Scientific Research on Intractable Diseases, “Research on Intractable Inflammatory Bowel Disorders”.
- In severe cases, surgery should always be considered a treatment option, and medical treatment should be carried out in close communication with the surgeon (please refer to Figs. 3, 4, 5, 6, 7, 8 and 9).

## 5-Aminosalicyclic acid (ASA)

**BQ 15. What are the benefits and precautions for the use of 5-ASA drugs in the treatment of IBD?**

### Statements

- 5-ASA is effective in inducing remission in active UC and preventing the relapse of UC in remission.
- The effect of 5-ASA on CD is generally lower than that on UC, and although 5-ASA has a suppressive effect on active CD, it has not been proven to be effective in maintaining remission.
- It should be kept in mind that there are cases of 5-ASA intolerance.

These statements and supplementary information were made with reference to [91–104].

### Supplementary information

Although 5-ASA drugs have relatively few side effects, they can cause abdominal pain, fever, joint pain, and bloody stools, making it appear as if the IBD itself is worsening. Saito et al. reported that the drug-induced lymphocyte stimulation test (DLST) has low sensitivity but high specificity for the diagnosis of 5-ASA allergy [103]. Therefore, in addition to 5-ASA allergy, there are cases of 5-ASA intolerance without DLST positivity. 5-ASA intolerance (allergy) should be suspected when the clinical course after the start of 5-ASA administration is unnatural.

**BQ 16. Are oral and topical 5-ASA formulations useful for the induction of remission in mild to moderately active UC?**

### Statements

- Oral and local 5-ASA formulations are useful for the induction of remission in mild to moderately active UC.
- The combination of oral and enteral 5-ASA formulations is useful when a more potent effect on mild to moderately active distal UC is needed.

These statements were made with reference to [93, 105–108].

**BQ17. Is maintenance treatment with 5-ASA drugs for UC in remission useful in maintaining clinical and endoscopic remission?**

### Statement

- Maintenance treatment with 5-ASA drugs for UC in remission is useful in maintaining clinical and endoscopic remission.

This statements was made with reference to [92, 94].

**BQ18. Are 5-ASA suppositories useful for the induction of remission in mild to moderately active UC of the proctitis type?**

### Statement

- 5-ASA suppositories are useful for the induction of remission in mild to moderately active UC of the proctitis type.

This statement was made with reference to [109–111].

**CQ3. What is the appropriate maintenance dose of 5-ASA for treating UC?**

### Recommendation

- It is recommended that the dose of 5-ASA in the maintenance therapy for UC be 2 g or more. **[Strong recommendation, high-quality evidence]**

### Commentary

The latest Cochrane analysis [94] shows that the administration of 2 g or more of 5-ASA is effective for maintaining remission. There was no difference in efficacy between the various 5-ASA preparations. Regarding pH-dependent mesalazine delayed-release preparation (Asacol^®^), it has been shown that there is no statistically significant difference in the efficacies of maintaining remission between 1.2 g/day and 2.4 g/day and between 2.4 g/day and 4.8 g/day [112, 113]. However, for patients aged less than 40 years or those with total colitis, it has been reported that the administration of 4.8 g/day of Asacol^®^ significantly increased the remission rate and duration of remission compared with the administration of 2.4 g/day of Asacol^®^ [113]. For time-dependent mesalazine controlled-release preparations (Pentasa^®^), it has been reported that there is no statistically significant difference in the efficacies of maintaining remission between 1.5 g/day and 3.0 g/day [114]. The dose range for maintaining remission in Multi Matrix System™ (MMX) mesalazine has not yet been investigated.

**CQ 4. Does 5-ASA treatment reduce the risk of UC-associated colorectal cancer (CRC)?**

### Recommendation

- It is recommended that 5-ASA treatment reduces the risk of UC-associated CRC. **[Strong recommendation, low-quality evidence]**

### Commentary

One of the risk factors for the development of UC- associated CRC is the length of the disease period [115], and successful treatment of UC is thought to reduce the risk of UC-associated CRC [116]. Several meta-analyses have demonstrated the protective effect of 5-ASA on IBD-associated CRC [117–119]; however, there is also a report that 5-ASA does not prevent the development of IBD- associated CRC [120]. Recently, Bonovas et al. [121] conducted a comprehensive meta-analysis of 31 studies and reported that exposure to 5-ASA reduces the risk of developing IBD-associated CRC to 43%. Twenty-one of these studies had limited data on UC but showed a 50% reduction in the risk of UC-associated CRC associated with 5-ASA use. In addition, four studies were analyzed to determine whether mesalazine is effective on carcinogenesis in patients receiving mesalazine at doses above 1.2 g/day. As a result, mesalazine doses above 1.2 g were more prominent in reducing colonic neoplasia risk. However, the analysis of two reports describing doses of less than 1.2 g/day showed no significant effect. Furthermore, since it has been reported that inflammation itself is an independent risk factor for the development of CRC in UC patients [122], the anti-inflammatory effect of 5-ASA is also considered to be involved in reducing the risk of UC-associated CRC.

**FRQ 1. Is the combined use of 5-ASA with biologics or immunomodulators (IMs) for CD in remission useful?**

### Statement

- The combination of 5-ASA with biologics or IMs may be considered as a maintenance therapy for CD in remission, but its usefulness has not been proven.

### Commentary

5-ASA drugs have not been shown to be more effective than the placebo group in 2010 and 2016 meta-analyses of the maintenance of remission after induction therapy [100]. In addition, the American College of Gastroenterology (ACG) Clinical Guideline states that oral 5-ASA drugs have not been demonstrated to be effective in maintaining CD remission and are not recommended for long-term treatment [75].

The integrated analysis of nine randomized controlled trials (RCTs) in a 2011 Cochrane Review demonstrated that the effect of 5-ASA preparations on maintaining remission in CD patients after surgical treatment as an induction therapy was found to be slightly significant in suppressing recurrence compared to the effect of placebo group [101]. At present, the role of the use of 5-ASA alone is often negative for maintaining CD remission but the additive and synergistic effects of the combined use of 5-ASA with biologics or IMs for CD in remission are unclear.

## Nutritional therapy

**BQ 19. What are the benefits and caveats of nutritional therapy in the treatment of IBD?**

### Statements

- It is not clear that nutritional therapy, such as enteral nutrition and central venous nutrition alone, effectively induces remission in UC. Nevertheless, drug therapy and blood-cell removal therapy should be the mainstay of UC treatment.
- Enteral nutrition is an effective remission-inducing therapy for active CD. Enteral nutrition thrapy is safe, but acceptance of the treatment can be difficult.
- Home enteral nutrition is effective in maintaining the remission of CD.

These statements were made with reference to [3, 16, 26, 123–132].

## Cytapheresis

**BQ 20. What are the benefits and cautions of Cytapheresis (CAP) in the treatment of IBD?**

### Statements

- In patients with moderate to severely active UC, blood-cell removal therapy should be considered a treatment option.
- Twice-weekly intensive therapy can reduce the time to the initiation of remission and improve the remission rate compared to once-weekly treatment in both UC and CD.
- In active CD, patients with colorectal lesions, granulocyte and monocyte adsorption apheresis (GMA) should be considered in combination with pharmacological and nutritional therapy when they are ineffective or inapplicable.

These statements and supplementary information were made with reference to [133–145].

### Supplementary information

Although CAP is a safe treatment with few side effects, it is difficult to perform in patients in whom access to peripheral blood vessels is difficult (including patients with dehydration and anemia) because effective blood flow cannot be obtained. In addition, it is known from clinical practice that the therapeutic effect of CAP is poor in severe cases of UC, such as those with extensive ulcerations. Although it is not covered by insurance, there is a report on the effect of CAP on maintaining the remission of UC [141], and there is only one case report on the effect of CAP on maintaining the remission of CD [142].

## Immunosuppressive drugs and biologics

**BQ 21. What are the precautions to take when using immunosuppressive drugs?**

### Statements

- The use of overlapping immunosuppressive therapy should be done with caution, taking into account the risk of infection and other risks.
- In patients infected with hepatitis B virus (HBV) (carriers and those previously infected), the risk of developing hepatitis B due to the HBV reactivation should be considered after the initiation of immunosuppressive drugs.
- Anti-TNF-α antibody therapy should be used with caution because of the risk of tuberculosis complications.

These statements were made with reference to [146, 147].

## Corticosteroids

**BQ 22. What is the usefulness and precautions for the use of corticosteroids in IBD treatment?**

### Statements

- Corticosteroids have potent anti-inflammatory effects and are useful for inducing the remission of UC and CD.
- Corticosteroids are not useful for maintaining remission because of long-term administration side effects.
- After the initiation of corticosteroid therapy, it is preferable to reduce the dose to less than 10 mg/day of prednisolone (PSL) equivalent (3 mg/day for budesonide) within three months.

These statements were made with reference to [148–154].

**BQ 23. Is budesonide foam useful for UC?**

### Statement

- Budesonide enema is useful for mild to moderate UC from the rectum to the sigmoid colon.

This statement was made with reference to [155–157].

**BQ 24 Are steroids (PSL, budesonide) useful for induction therapy for CD?**

### Statements

- Budesonide is useful for mildly to moderately active ileocecal CD (ileum to ascending colon).
- The Administration of oral steroids (PSL 40 mg/day) is useful for moderately to severely active ileocecal CD
- Intravenous administration of steroids (PSL 40–60 mg/day) is effective for severely active CD after excluding infections.
- The administration of steroids is involved in the development of perforating complications (abscesses and fistulas), therefore, it is generally contraindicated in such cases.

These statements were made with reference to [75, 148, 149, 153, 158–161].

## Immunomodulators

**BQ 25. What are the usefulness and precaution for the use of immunomodulators in the treatment of IBD?**

### Statements

- Azathioprine (AZA)/6-mercaptopurine (6-MP) treatment effectively prevents relapse in UC in remission, especially in steroid-dependent patients and those unable to maintain remission with 5-ASA.
- AZA/6-MP treatment is effective in maintaining remission for CD patients who achieve remission.
- Adverse effects of AZA/6-MP treatment include nausea and other GI symptoms, myelosuppression, alopecia, and pancreatitis.

These statements were made with reference to [162–180].

**CQ 5. Is the NUDT15 gene R139C variant useful for predicting acute severe leukopenia induced by thiopurine?**

### Recommendation

- The *NUDT15* gene R139C variant is useful for predicting acute severe leukopenia and severe alopecia induced by thiopurine, and we recommend confirming whether the *NUDT15* gene R139C variant is present before the initiation of thiopurine. **[Strong recommendation, high-quality evidence]**

### Commentary

In 2014, a study from Korea revealed that a single-nucleotide polymorphism in the nucleoside diphosphate-liked moiety X-type motif 15 (*NUDT15*) gene, in which C at position 415 changes to T, was strongly associated with acute severe leukopenia induced by thiopurines [174]. This polymorphism converts the 139th amino acid from arginine (Arg; R) to cysteine (Cys; C) (R139C variant). This was also confirmed in Japanese patients [176]. Acute severe leukopenia is inevitable in patients homozygous for the *NUDT15* gene R139C variant. These patients also develop severe alopecia. In a large multicenter study conducted in Japan involving 1,291 patients previously treated with thiopurine (the MENDEL study), all 49 patients homozygous for the *NUDT15* gene R139C variant discontinued thiopurine early due to adverse events (AEs) [181]. Among Japanese individuals, the frequency of homozygotes (Cys/Cys) for the *NUDT15* gene R139C variant is approximately 1%, and the frequency of heterozygotes (Arg/Cys) is approximately 20%.

**CQ 6. Is thiopurine effective for the prevention of postoperative recurrence of CD?**

### Recommendation

- We propose the use of thiopurines for the prevention of postoperative recurrence of CD. **[Weak recommendation, moderate-quality evidence]**

### Commentary

Thiopurines, such as azathioprine (AZA) and 6-mercaptopurine (6-MP), are more effective than placebo in preventing postoperative clinical recurrence of patients with CD [166, 182, 183]. However, whether thiopurine is superior to placebo in preventing endoscopic recurrence after surgically induced remission in CD is controversial [166, 183]. The superiority of thiopurines over 5-ASA compounds in preventing postoperative recurrence of CD has not yet been established [183, 184]. Further studies are needed to (i) compare the efficacy between thiopurines vs. molecular-target drugs (e.g., TNF antagonist), and (ii) determine whether thiopurines on molecular-target drugs provide an additional effect, in preventing postoperative recurrence of CD.

Note: Currently, 6-MP is not covered by insurance for the treatment of IBD in Japan.

**FRQ 2. Is thiopurine associated with an increased incidence of lymphoma in Asian IBD patients?**

### Statement

- The risk of developing lymphoma associated with thiopurine may be lower in Asian IBD patients than in Caucasian patients, but further studies are warranted.

### Commentary

A large French cohort study reported that the incidence rate of lymphoproliferative disease was 0.90/1000 patient-years (95% confidene interval (CI) 0.50–1.49) in patients receiving thiopurine compared with 0.26/1000 patient-years (95% CI 0.10–0.57) in patients not receiving thiopurine. The adjusted hazard ratio was 5.28 (95% CI 2.01–13.9) [169]. No prospective cohort study has examined the incidence of lymphoma associated with thiopurine in Asian IBD patients. A Korean retrospective cohort study reported a standardized incidence ratio for lymphoma of 2.03 (95% CI 0.81–4.18) for all patients versus 5.93 (95% CI 6.16–15.18) for patients receiving thiopurine [185]. A questionnaire survey in Japan reported that 28 out of 36,939 patients had hematological malignancies, including 10 patients with lymphoma and the odds ratios for the incidence of hematological malignancies associated with thiopurine were 1.37 (95% CI 0.30–6.24) for UC and 1.86 (95% CI 0.60–5.78) for CD [172]. On the other hand, a recent Japanese study using a nationwide administrative database reported no increase in the incidence of lymphoma associated with thiopurine in patients with IBD [186].

## Calcineurin inhibitors

**BQ 26. What are the benefits and precautions for the use of calcineurin inhibitors in the treatment of IBD?**

### Statements

- Consider the use of intravenous cyclosporine (CsA) for severe UC that does not respond to steroid therapy.
- Oral tacrolimus should be considered for patients with severe UC who do not respond to steroids.

These statements were made with reference to [187–192].

**FRQ 3. Is tacrolimus treatment effective for CD?**

### Statement

- Tacrolimus treatment is useful for patients with CD, but further research results will need to be accumulated.

### Commentary

Few RCTs have reported the efficacy and AEs of tacrolimus (Tac) in patients with refractory CD, and only case studies have been reported [193, 194]. Recently, a systematic review and meta-analysis of the therapeutic effects and AEs of Tac in patients with CD was conducted [195]. Based on the case studies, systematic reviews and meta-analyses to date, Tac therapy is effective for patients with CD, and Tac therapy can be considered an option for patients with active CD. However, few RCTs have been conducted to accurately evaluate the efficacy of this therapy, and further clinical studies are needed.

Note: Currently, Tac is not covered by insurance for the treatment of CD in Japan.

## Anti-TNF-α agents

**BQ 27. What are the benefits and precautions for the use of anti-TNF-α agents in the treatment of IBD?**

### Statements

- Anti-TNF-α agents effectively induce and maintain remission in moderately to severely active UC that is either steroid-resistant or steroid-dependent.
- Anti-TNF-α agents are effective in inducing and maintaining the remission of pro-inflammatory CD.

These statements were made with reference to [2, 20, 146, 196–210].

**BQ 28. Is there a difference in the effectiveness of Infliximab (originator) and biosimilars in introducing and maintaining remission?**

### Statement

- There is no difference in the effectiveness of Infliximab (originator) and biosimilars in inducing and maintaining remission.

This statement and supplementary information were made with reference to [211–213].

### Supplementary information

While the use of CT-P13 should be considered from the viewpoint of medical cost-effectiveness, the nocebo effect that occurs when switching to a biosimilar (the effect of treatment that should have been obtained is affected due to anxiety and poor impression about the use of biosimilar on the part of patients) has become an issue [211–213]. In this regard, physicians and other healthcare professionals (nurses and pharmacists) should carefully explain the use of biosimilar when prescribing them to patients.

**CQ 7. Is retreatment with the same anti-TNF-α agent effective and safe for the relapse after one discontinuation?**

### Recommendation

- Retreatment with the same anti-TNF-α agent for the relapse after discontinuation is recommended because it is generally effective and safe. **[Weak recommendation, low-quality evidence]**

### Commentary

The efficacy of retreatment with the same anti-TNFα agent for the relapse after the discontinuation is generally favorable (> 80%) [214–218]. However, careful interpretation is required because these retrospective observational studies might have different efficacy criteria and most reports focused more on IFX than on other anti-TNFα agents.

There may be a concern that anti-drug antibodies could be increased due to the interval of drug suspension. However, drug suspension during remission is generally considered safe because fewer anti-drug antibodies are produced during drug suspension than with intermittent administration aimed at inducing remission [214]. On the other hand, it has also been confirmed that the presence of anti-drug antibodies is a risk factor for an infusion reaction during re-administration [216]. There is no evidence for the efficacy of switching to another anti-TNFα agent with less immunogenicity upon relapse.

**CQ 8. Is concomitant use of immunomodulators and anti-TNF-α agents useful in the treatment of patients with IBD?**

### Recommendations

- In the treatment of CD, combination therapy is recommended because it is more effective than monotherapy, which each drug is used separately. **[Strong recommendation, high-quality evidence]**

- In the treatment of UC, combination therapy is suggested, because it may be more effective compared to monotherapy, which each drug is used separately. **[Weak recommendation, moderate-quality evidence]**

### Commentary

In the SONIC study, patients with moderate to severe CD who had not previously been treated with biologics or immunomodulatory drugs were assigned to three groups, namely, a IFX monotherapy group, an immunomodulator (IM) monotherapy group, and a group treated with the combination of both therapies. The findings showed that the combined therapy group was superior to the other two groups in terms of remission induction and endoscopic healing rates [200]. In the DIAMOND study performed in Japan, the efficacy of a combination therapy using adalimumab (ADA) with IMs in comparison with ADA monotherapy was examined, but the remission rates were not significantly different between two groups [203]. However, a subanalysis report showed that the endoscopic efficacy tended to be greater when combination therapy was used [219]. Based on these results, meta-analyses and major guidelines published in Western countries strongly recommend the concomitant use of IMs when using anti-TNF-α antibody agents in CD patients [75, 159, 220]. The additional efficacy of combination therapy is based on increasing concentrations of anti-TNF-α antibody products as a result of the suppression of the production of anti-drug antibodies [75, 220]; continuing the combination therapy for the first 6 months is considered particularly important [159].

In UC patients, the only one major research study, the UC SUCCESS trial, examined combination therapies using IFX with IMs [221]. In this trial, patients with moderate to severe UC who had not previously been treated with biologics or IMs were assigned to the following three groups: an IFX monotherapy group, an IM monotherapy group, and a group treated with a combination of both therapies. The findings showed that the combination therapy group was superior to the other two groups in terms of the rates of steroid-free induction of remission and endoscopic healing. ADA and golimumab, which are less immunogenic than IFX, have not yet been shown to increase the effect of the combination therapy. For this reason, the efficacy of combination therapy is limited to IFX, and even guidelines in Western countries do not recommend it as strongly as in CD [222, 223].

Given that combination therapy can also be associated with risks, there needs to be a comprehensive assessment that includes an estimation of each individual patient's clinical background, such as age, comorbidity, and medical history, as well as an assessment of the need for combination therapy depending on the course of treatment. Decisions for the administration of combination therapy and its duration should be based on the abovementioned assessment findings [224].

**CQ 9. Do anti-TNF-α agents prevent the recurrence of CD after surgery?**

### Recommendation

- It is recommended that anti-TNF-α agents be administered to prevent endoscopic recurrence. **[Strong recommendation, moderate-quality evidence]**

### Commentary

We performed a systematic review and meta-analysis of a total of 570 patients, including 254 in an intervention group and 316 in a control group [225]. The results of eight randomized control studies performed to determine efficacy of anti-TNF-α agents administered after surgery for the prevention of endoscopic recurrence [relative risk (RR) 0.34, 95% CI 0.22–0.53] showed no increase in AEs (RR 1.75, 95% CI 0.81–3.79). However, clinical recurrence was not prevented (RR 0.60, 95% CI 0.36–1.02) [225].

The meta-analysis included analyses conducted during 1–2 years of treatment with anti-TNF-α agents after surgery, without the consideration of concomitant treatment and with different definitions for outcomes. Moreover, the participants included patients who had been treated with an anti-TNF-α agent and those naïve to anti-TNF agent before surgery. The findings of efficacy may differ based on the outcome of interest, such as long-term prevention, the avoidance of further surgery, cost-effectiveness, and safety. Our results provide evidence for the efficacy as well as the safety of anti-TNF-α agents, which should be confirmed in a future nationwide observational or prospective study.

**CQ 10. Is the long-term combination of anti-TNF-α agents and immunomodulaors safe?**

### Recommendation

- It is recommended to evaluate the long-term combination of anti-TNF-α agents and immunomodulaors from the viewpoint of usefulness and safety, considering the patient background, treatment course, and risk differences between Japan and Western countries. **[Strong recommendation, moderate-quality evidence]**

### Commentary

The combination therapy of anti-TNFα agents and immunomodulaors (IMs) has been reported to increase the risk of opportunistic infections such as herpes zoster, lymphoma (non-Hodgkin) and skin cancer (nonmelanoma) in Western countries [159, 169, 226–234]. However, studies in Japan did not confirm an increase in the incidence of lymphoma associated with the combination therapies [172, 186]. Regarding skin cancer, a 3.39 to 4.03-fold increase in risk was reported in Japanese patients treated with IMs, and the actual prevalence was only 2.94 to 4.94 per 100,000 per year, which is very low compared with that in the Western population.

Long-term combination therapies in young to adolescent male patients have been reported to increase the risk of high-mortality hepatosplenic T-cell lymphoma in the Western countries, but not in Japan [210, 235]. Regarding the contribution of IMs to Epstein-Barr (EB) virus infection, it has been reported that the incidence of lymphoma increases in preinfected patients and the risk of hemophagocytic syndrome and lymphoma increases in uninfected patients when they are first infected. Therefore, it is recommended to test for EB virus serum antibodies prior to administration and to avoid the use of IMs in uninfected individuals. Screening for cervical cancer is recommended for female patients receiving long-term AZA or 6-MP therapy [210].

At present, there is no previous report that provides a clear answer as to how long the combination anti-TNFα agents and IMs will increase risks. Despite the benefit of IMs such as the suppression of antibody production for anti-TNFα agents, the indication is determined by considering the risks and benefits for each patient.

**FRQ 4. Can anti-TNF α agents be stopped?**

### Statement

- The discontinuation of anti-TNFα agents should be carefully discussed, considering the risk of relapse, safety, medical costs, and patient desire, because discontinuaiton is likely to increase the risk of relapse.

### Commentary

The possibilities of discontinuating anti-TNFα agents in IBD patients maintaining long-term remission with the maintenance treatment are increasingly discussed. However, it is necessary to take not only the advantages but also the disadvantages of long-term treatment, such as safety, medical costs, and treatment fatigue, into consideration when discussing the discontinuation.

The clinical outcomes after the discontinuation of anti-TNFα agents in CD differed slightly from those in UC. A prospective study (the STORI trial) was conducted for CD patients in steroid-free remission with a combination therapy of IFX and IM for 6 months, and the discontinuation of IFX resulted in relapse in approximately half of the patients. Male sex, a history of surgery, anemia, inflammatory response, and elevated fecal calprotectin were identified as risk factors for relapse [202]. There have been many retrospective analyses in both UC and CD. The 1-year relapse rate has been reported to be approximately 20–40% when anti-TNFα agents are stopped [215, 217, 236, 237].There have been a few reports regarding the usefulness of maintenance therapy with IMs after the discontinuation of anti-TNF agents [215, 217], while there is no consensus on the risk factors for relapse in UC.

**FRQ 5. Are anti-TNF-α agents useful for the treatment of internal fistulas in CD?**

### Statement

- There is little evidence to justify the selection of anti-TNFα agents for the treatment of all internal fistulas in CD; thus, a comprehensive assessment is required for each case, including surgical treatment.

### Commentary

Few high-evidence-level reports have served as a basis for this FRQ. Anti-TNFα agents were largely less effective on internal fistulas than on external fistulas [238, 239], and the ACCENT II study of IFX also showed a 45% fistula closure rate at 14 weeks in 25 patients with rectovaginal fistulas [240]. The study conducted in 20 Japanese institutions was reported, in which 93 cases of internal fistulas (77% of the cases were small-bowel-to-small-bowel or enterocolic fistulas, 17% were enterovesical fistulas, and 5% were enterovaginal fistulas) were treated with either IFX or ADA. The results of the study showed a cumulative surgery rate of 47% during a nearly 4-year observation period and a fistula closure rate of 27% at 5 years after the initiation of anti-TNFα agents. The findings also revealed that low disease activity (p = 0.017) and a shorter time interval between the diagnosis of fistula and the administration of an anti-TNFα agents (p = 0.048) were factors associated with the avoidance of surgery [241].

To determine the therapeutic approach for internal fistulas, the condition of the fistula must first be confirmed through diagnostic imaging; each case should be assessed comprehensively to determine the treatment strategy.

**FRQ 6. Is an anti-TNF-α agent useful for CD with GI bleeding?**

### Statement

- An anti-TNF-α agent is one option for CD with GI bleeding.

### Commentary

Massive bleeding from the GI tract is rarely seen in CD. First, we start conservative treatment with fasting and fluid replacement, and then the intestinal tract is rested. There have been reports that steroids and IFX were effective as drug treatments [242, 243]. However, no reports have summarized a large number of cases. It has also been reported that IMs reduce the risk of bleeding from lower GI lesions [244]. Endoscopic hemostasis should be attempted if possible. There are reports that vasopressin injection and arterial embolization have been shown to be useful in angiography [245, 246], but intestinal necrosis due to intestinal ischemia has been a problem with arterial embolization. Surgical treatment is required when hemostasis is difficult to achieve with medical treatment [247]. The surgical rate for initial massive bleeding has been reported to be 20–90%, and the surgical rate for rebleeding after conservative treatment has been reported to be 30–35% [248, 249].

## Ustekinumab

**BQ 29. Is ustekinumab useful for treating CD?**

### Statement

- Ustekinumab is useful as an induction/maintenance therapy for moderate to severe CD.

These statements were made with reference to [250–255].

**FRQ 7. Is the concomitant use of an immunomodulator with ustekinumab more useful than ustekinumab monotherapy as an induction therapy for CD?**

### Statement

- Based on the current evidence, we cannot state that the concomitant use of an immunomodulator with ustekinumab is more useful than ustekinumab monotherapy as an induction therapy for CD.

### Commentary

At the time of the survey, there have been no RCTs directly comparing the effectiveness of ustekinumab (UST) monotherapy and UST plus an immunomodulator (IM) as an induction therapy for CD. Six studies that analyzed induction therapy with UST for CD patients were extracted [250, 252, 256–259], and a meta-analysis was performed. The concomitant use of IM was significantly more effective than UST monotherapy (OR: 1.35, 95% CI: 1.06–1.71) [260]. However, the risk of bias was considered to be high and the level of evidence was judged to be “weak”. There is currently no clear evidence of AEs with the addition of an IM to UST [261], however, the risks of infection and malignancy should be considered as with the risks of using IMs in combination with other biological agents. Collectively, the current evidence in the published papers does not indicate that the concomitant use of an IM with UST is more useful than UST monotherapy as an induction therapy for CD, and accumulating evidence is necessary in the future.

**FRQ 8. Is ustekinumab useful for preventing postoperative recurrence in CD?**

### Statement

- There have been no reports investigating the prevention of postoperative recurrence of CD by ustekinumab.

## Commentary

There have been many reports on the usefulness of anti-TNF-α agents for the prevention of postoperative recurrence in CD [262, 263]. UST was approved for CD worldwide from 2016 to 2017, and there has been no report investigating its effect for the prevention of postoperative recurrence at the time of the survey (April 2020). Data will be collected in the future regarding the selection of drugs suitable for preventing postoperative recurrence.

**FRQ 9. Is ustekinumab useful for perianal lesions of CD?**

### Statement

- Ustekinumab may be useful for perianal lesions in CD, and further evidence needs to be accumulated.

### Commentary

As of April 2020, there have been no prospective studies investigating the efficacy of UST for perianal lesions in CD. According to the summarized subanalysis limited to patients with active penetrating lesions in placebo-controlled RCTs, 39 of 150 patients (26%) showed improvement in penetrating lesions 8 weeks after UST treatment, and the improvement rate was higher than that for placebo at 8, 22, and 44 weeks [264]. In addition, a meta-analysis of these subanalyses showed a risk ratio of UST to improvement of penetrating lesions to placebo of 1.77 (95% CI: 0.93–3.37), suggesting a nonsignificant but improving effect of UST [238]. The real-world data after approval are almost in line with the results of clinical trials [256, 258, 265].

Based on these results, UST may be useful for perianal lesions. However, the subanalysis of the clinical trials includes all penetrating lesions other than perianal disease, and there remain many unclear points, such as in what kind of anal lesions is UST effective and how many bionaive cases are included. Detailed analysis focusing on perianal lesions is required in the future.

**FRQ 10. Is ustekinumab safe for pregnant women with CD?**

### Statement

- The safety of ustekinumab to pregnant women with CD has not been established.

### Commentary

In approximately 10 cases of CD reported so far, serious problems were not reported in either mothers or infants, except for one case of miscarriage at 8 weeks gestation [266]. UST, a human monoclonal IgG1 antibody, can transfer to the placenta in late pregnancy and to breast milk after parturition [267]. In two CD patients who used UST until late pregnancy, cord-blood drug levels were higher than maternal serum levels, but there was no problem with the condition of the children in either case [268, 269]. In addition, the transfer of UST to breast milk has been reported [269], but there has been no report that there is a major problem in the growth process of the infants. However, the safety of UST for pregnant women has not been established due to the limited data, and the possibility of continuing administration should be carefully examined in each individual case.

## Vedolizumab

**BQ 30. Is vedolizumab effective for UC?**

### Statement

- Vedolizumab is effective in inducing and maintaining remission in moderate to severe UC.

This statement was made with reference to [111, 270–280].

**BQ 31. Is vedolizumab effective for CD?**

### Statement

- Vedolizumab is effective in inducing and maintaining remission in moderate to severe CD.

This statement was made with reference to [275, 276, 281–287].

**BQ 32. Is vedolizumab effective for IBD patients refractory to anti-TNFα agents?**

### Statements

- Vedolizumab is useful for both UC and CD patients refractory to anti-TNFα agents.
- Vedolizumab is particularly useful for maintaining remission in UC and CD patients refractory to anti-TNF-α agents.

These statements were made with reference to [273, 274, 277, 283, 285, 288–293].

**BQ 33. What should we consider in the safety of vedolizumab?**

### Statements

- We need to pay attention to respiratory tract infections (especially upper respiratory tract infections) and enteric infections (e.g., *C. difficile*) during vedolizumab treatment.
- No significant association of vedolizumab with the development of progressive multifocal leukoencephalopathy (PML) or malignancy has been reported to date.
- The safety of vedolizumab for pregnant women, lactating women, women attempting to conceive, and children has not been sufficiently established.

These statements were made with reference to [276, 294–304].

**FRQ 11. Are anti-TNFα agents effective for patients refractory to vedolizumab?**

### Statement

- Evidence regarding the efficacy of anti-TNFα agents in IBD patients in whom vedolizumab failed has not been accumulated, and this issue remains to be investigated.

### Commentary

A post-hoc analysis of the clinical trials of vedolizumab (VDZ) demonstrated that VDZ was less effective in patients in whom anti-TNFα antibody agents failed than in those who had not received anti-TNFα agents in both UC and CD [273, 282]. On the other hand, we did not find any studies investigating the efficacy of anti-TNFα agents in patients in whom VDZ had failed.

**FRQ 12. Should vedolizumab be used with immunomodulators in IBD patients?**

### Statement

- The currently available evidence does not suggest a benefit for the concomitant use of immunomodulators with vedolizumab, but further studies are warranted.

### Commentary

There have been no RCT to examine the efficacy of the concomitant use of VDZ with IMs. A subgroup analysis of the GEMINI 1 study, a phase III study of VDZ in UC patients, reported that the rates of clinical remission and mucosal healing at weeks 6 and 52 were higher in patients receiving VDZ than in those receiving placebo regardless of use of IMs at baseline [305]. Most observational studies have reported that concomitant use of IMs did not affect the effectiveness of VDZ in patients with UC or CD [277, 293], while a small observational study reported that concomitant use of IMs was a predictor of clinical remission and clinical response at week 54 in patients with CD (odds ratio 8.33, 95% CI 2.15 -32.26) [306]. It has been reported that concomitant use of IMs does not affect serum trough concentrations of VDZ or the development of anti-VDZ antibodies [294, 307]. In terms of safety, an integrated analysis of the clinical trials of VDZ reported that use of IMs at baseline was not associated with serious infections [294]. On the other hand, a retrospective study demonstrated that concomitant use of immunosuppressive therapy (IMs or steroids) was associated with infections (odds ratio 1.72, 95% CI 1.20 -2.46) [308].

## Tofacitinib

**BQ 34. Is tofacitinib useful for refractory patients with moderate to severely active UC?**

### Statement

- Tofacitinib is useful for refractory patients with moderate to severely active UC.

This statement was made with reference to [309, 310].

**BQ 35. What are the precautions to take when using tofacitinib in UC treatment?**

### Statements

- Tofacitinib therapy should be used with caution for the risk of infection complications, especially herpes zoster infections.
- Elderly patients with cardiovascular disease and rheumatoid arthritis treated with tofacitinib (predominantly 10 mg twice daily) have an increased risk of pulmonary embolism.

These statements were made with reference to [311–315].

**CQ 11: Is tofacitinib effective for UC patients refractory to anti-TNFα agents?**

### Recommendation

- It is recommended to use tofacitinib for UC patients refractory to anti-TNFα agents **[Weak recommendation, moderate-quality evidence]**

### Commentary

Tofacitinib 10 mg twice daily was shown to be more effective as induction therapy in a phase III, randomized, double-blind, placebo-controlled trial; remission at 8 weeks occurred in 12.6% of the patients in the tofacitinib group versus 1.5% in the placebo group (P < 0.01) in the OCTAVE 1 trial and in 12.0% versus 0.0% (P < 0.01) in the OCTAVE 2 trial [309]. In the maintenance OCTAVE Sustain trial, week 52 remission rates were 36.6% in the tofacitinib group versus 12.0% in the placebo group(p < 0.0001). A network meta-analysis comparing tofacitinib with other drugs also demonstrated its usefulness in patients who had failed to respond to anti-TNFα therapy [316].

## Endoscopic treatment for CD

**CQ 12. Can endoscopic balloon dilation (EBD) for intestinal stenosis in CD avoid surgical intervention?**

### Recommendation

- EBD is recommended for indicated intestinal stenosis in CD because it can avoid surgical intervention at least in the short-term. **[Strong recommendation, moderate-quality evidence]**

### Commentary

Indications for EBD for intestinal stenosis in CD include (a) intestinal stricture length of 5 cm or less, (b) no fistula or abscess in stricture site, (c) no deep ulcer in stricture site, and (d) no severe curvature and strong adhesion in strictured part [317]. It is important to take into carefully consider the abovementioned indications when performing EBD in CD patients. In several meta-analyses with regard to EBD for intestinal stenosis in CD focusing on lesions of the lower GI tract (small intestine, ileocolonic anastomosis, large intestine), the short-term technical success rate was 86–94%, and the short-term clinical symptom improvement rate was 58–87% [318–321]. For upper GI (gastroduodenal) lesions, a meta-analysis and a prospective observational study reported a short-term technical success rate of 93–100% and a short-term clinical symptom improvement rate of 87% [322, 323]. Since complications associated with EBD for intestinal stenosis in CD are reported in 2 to 6% and perforation is also observed in 1 to 3%, strict intraoperative and postoperative monitoring and management are needed [318–323].

## Surgical treatment and colitic cancer

**BQ 36. What are the indications and precautions for the surgical treatment of IBD?**

### Statements

- In severe cases of IBD and those with cancer or dysplasia, surgical treatment is expected to improve life expectancy. In addition, the quality of life can be improved in patients with symptoms caused by the primary disease that do not improve with medical treatment, side effects of medication, and extraintestinal complications (especially pyoderma gangrenosum).
- Postoperative complications such as suture failure and intestinal obstruction, ileocolitis in UC, and short bowel syndrome in CD can occur.
- Complications such as significant bleeding and toxic megacolon are likely to occur in elderly patients with IBD due to the delay in surgery.

These statements were made with reference to [16, 26, 111, 324–338].

**CQ 13: Who should receive surveillance colonoscopy for detecting CRC in UC?**

### Recommendation

- Surveillance colonoscopy is useful and recommended for patients with total and left-sided UC starting at 8 years after the onset of UC. **[Strong recommendation, low-quality evidence]**

### Commentary

Long-standing UC is a well-known risk factor for the development of colorectal cancer (CRC). A Cochrane Review integrating four observational studies showed that surveillance colonoscopy is associated with a reduction in CRC development and death [339]. This review included both CD and UC cases, and the CRC death rates were 7.7% and 22.3% in the surveillance and the nonsurveillance group, respectively. A multicenter retrospective study from Japan investigating surgically resected cases also demonstrated a survival benefit of the surveillance colonoscopy [33]. Western guidelines recommend that patients with total and left-sided colitis receive surveillance colonoscopy starting at eight years after the onset of UC [223, 340]. However, approximately 20% of CRC developed within eight years of UC duration [33, 341]. Especially in late-onset UC cases (> 40 or > 50 years old), the concomitant CRC is high even within 8 years of UC duration [33, 342]. Patients with primary sclerosing cholangitis (PSC) should receive surveillance colonoscopy from the onset of the disease due to its ambiguous disease onset and high CRC risk [223, 340]. The optimal interval of surveillance colonoscopy has not been established. Several studies have showed that those who received surveillance within two or three years had a better survival [33, 343]. Although European Crohn’s and Colitis Organisation (ECCO) guidelines set a different interval from 1 to 5 years according to the risk stratification, its evidence level is not high [340]. Analyses from surgically resected cases showed that 12% of CRC cases were already staged III or IV at the time of surgery among patients who received surveillance within two years [33]. A determination of the optimal surveillance interval incorporating both the CRC risk and progression speed is warranted.

**CQ 14. What kind of biopsy method is recommended for UC-associated CRC surveillance?**

### Recommendation

- Targeted biopsy is recommended for UC-associated CRC surveillance. **[Strong recommendation, moderate-quality evidence]**

### Commentary

An RCT demonstrated that targeted biopsy is comparable to random biopsy in terms of the neoplasia detection rate in UC-associated cancer surveillance [344]. Therefore, a targeted biopsy is recommended for patients with quiescent disease as enrolled in the RCT. On the other hand, there are cases with invisible dysplastic lesions that only random biopsy could detect [33, 345]. An observational study investigating 1000 consecutive cases of UC and CD showed that random biopsy increased the dysplasia detection rate by 15% [345]. The study mentioned that patients with the lead pipe appearance, PSC, and past dysplasia merit random biopsy. Random biopsy takes four samples every 10 cm, however, it is time-consuming and not realistic in clinical settings. Analyses from surgically resected specimens clarified the rectum and the sigmoid colon to be hotspots of UC-associated CRC in Japan [33]. Therefore, it is vital to carefully observe these hotspot regions and take biopsies from areas with subtle changes such as reddish (or pale) lesions and surface pattern changes in addition to targeted biopsy from suspicious lesions.

A meta-analytic study showed the benefit of chromoendoscopy over white-light endoscopy in UC-associated CRC surveillance [346]. When analyzing RCTs alone, although chromoendoscopy was superior to white-light endoscopy in the non-high-definition (HD) endoscopy setting, it was not evident in the HD endoscopy setting. Different RCTs adopted different concentrations of methylene blue or indigo carmine. In terms of narrow-band imaging (NBI), although a meta-analytic study denied the benefit of NBI over chromoendoscopy [347], a recent study using HD endoscopy showed a comparable neoplasia detection rate between NBI and white-light endoscopy in UC [348]. In real-world settings, it is vital to combine available modalities at each institution to increase the neoplasia detection rate.

**FRQ 13. How is cancer surveillance performed for CD?**

### Statement

- Cancer surveillance is recommended for CD patients. However, proper methods remain unclear.

### Commentary

European guidelines recommend surveillance colonoscopy for patients with CD, as the incidence of CRC is significantly higher in those than in the general population[349–352]. We performed a systematic review, and those results also confirmed that the standardized incidence ratios of colorectal and small-bowel cancer were significantly high in association with CD, although the incidence of anorectal lesion cancers was found to be significantly higher than that of either colonic or small bowel lesion cancers in Asian countries [353]. Therefore, we were not able to draw a conclusion regarding the usefulness of or provide a recommendation for surveillance colonoscopy for CD. Although efficacy has not been proven, an annual examination under anesthesia, as well as MRI and examinations by an expert IBD surgeon, are recommended for patients in Japan in the Japanese guidelines for intractable disease affiliated with the Japan Ministry of Health, Labor and Welfare.

**FRQ 14. Is endoscopic treatment recommended for colitis-associated cancer/dysplasia in patients with IBD?**

### Statement

- We cannot make a recommendation regarding endoscopic treatment for colitis-associated cancer/dysplasia.

### Commentary

Endoscopic treatment is recommended only for patients with a polypoid dysplastic lesion with a clearly visible boundary line, while close repeated follow-up surveillance colonoscopy examinations may also be conditionally needed [354]. However, long-term safety factors including recurrence, mortality, and morbidity related to endoscopic treatment have not been sufficiently investigated. In addition, the characteristics of metachronous or synchronous colitis-associated cancer have not been well elucidated. At present, we cannot recommend endoscopic treatment for colitis-associated cancer/dysplasia.

**FRQ 15. Are anti-TNF-α agents efficacious for pouchitis?**

### Statement

- Evidence for the efficacy of anti-TNF-α agents for pouchitis is limited.

### Commentary

The first-line treatment for pouchitis includes ciprofloxacin and metronidazole. Second-line treatment for those with antibiotic-resistant or dependent pouchitis has not been established. In real-world settings, drugs effective for UC are empirically used for pouchitis. One RCT investigated the efficacy of anti-TNF-α agents, comparing ADA with placebo [355]. This RCT failed to accrue 24 initially planned patients with pouchitis resistant to > 4 weeks of antibiotics, and enrolled only 13 patients. Thus, the study was underpowered to show the efficacy of ADA for antibiotic-resistant pouchitis. A meta-analytic study [356] integrated retrospective observational studies using anti-TNF-α agents, mainly one-arm studies consisting of a few cases. In this meta-analysis, the short-term (at ~ 8 weeks) clinical response rates of IFX and ADA were 56% (95% CI 36–75%) and 38% (95% CI 8–72%), respectively, while the long-term (at ~ 12 months) response rates of IFX and ADA were 59% (95%CI 45–72%) and 30% (95%CI 15–46%), respectively. This study also showed that those with CD of the pouch had a higher response rate to anti-TNF-α agents. Although anti-TNF-alpha agents seem useful for antibiotic-dependent or resistant-pouchitis based on the results of several observational studies, further studies with controls are warranted.

## Special situations

**BQ 37. How do you deal with elderly IBD patients?**

### Statements

- The treatment of elderly patients with IBD is essentially the same as that of patients of average age. Nevertheless, it is essential to determine the appropriate surgery timing in severe cases, bearing in mind that delays in diagnosis and surgery can have a prognostic impact.
- It is essential to consult a specialist as soon as possible in refractory cases resistant to immunosuppressive therapy.

These statements and supplementary information were made with reference to [16, 336, 337, 357–359].

### Supplementary information

There is no absolute definition of an elderly UC patient, but for convenience, an elderly UC patients is often defined as those “over 60” or “over 65”. The surgery rate remains the same in elderly UC patients as in nonelderly UC patients. However, it has been reported that the surgery rate of elderly-onset UC is higher than that of non-elderly-onset UC [359]. Therefore, it is necessary to clearly distinguish between elderly-onset UC with a short disease duration and young-onset UC with a long disease duration (aged UC).

**BQ 38. How do you deal with patients with IBD during pregnancy and lactation?**

### Statements

- Patients and their physicians should discuss and select a treatment for patients with IBD during pregnancy and lactation, taking into account each case's treatment benefits and harms.
- In many cases, the benefits of treatment outweigh medication harm; therefore, treatment should be continued during pregnancy.

These statements and supplementary information were made with reference to [360–376].

### Supplementary information

Although AZA, CsA, and Tac are contraindicated for administration to pregnant women in the Japanese package insert, no clinically significant teratogenicity or fetal toxicity has been demonstrated. Therefore, the above three drugs, with colchicine, can be administered with informed consent, even during pregnancy, under certain circumstances.

In clinical practice, anti-TNFα agents are administered to patients with moderate to severe IBD, and the continuation of administration is often necessary. When anti-TNFα agents are used beyond 22 weeks of gestation, live vaccines such as the Bacillus Calmette–Guérin vaccine (BCG) (usually administered at 5 to 7 months) should be avoided before the child reaches 6 months of age (until the disappearance of administered antibodies) [360].

**BQ 39. What are the extraintestinal complications observed in patients with IBD?**

### Statements

- Extraintestinal complications associated with IBD are mainly skin lesions and arthritis.
- Erythema nodosum and pyoderma gangrenosum are two of the most common skin lesions associated with IBD. Characteristically, they are often painful and can be relieved by controlling the inflammation of the intestinal tract.
- Arthritis associated with IBD includes ankylosing spondylitis and peripheral arthritis, both of which are negative for rheumatoid factors.

These statements were made with reference to [16, 377–382].

**CQ 15. Is thromboembolism prophylaxis necessary for hospitalized IBD patients?**

### Recommendation

- We propose that thromboprophylaxis in hospitalized IBD patients should be considered with an understanding of the increased risk of bleeding associated with the intervention. **[Weak recommendation, low-quality evidence]**

### Commentary

The risk of venous thrombosis in patients with UC and CD is reported to be approximately twice that of the non-IBD individuals [383] and is particularly higher during flares, the chronic active phase, and hospitaliztion periods [384]. Comorbidities and a history of steroid use are also associated with an increased risk of thrombosis [385]. Anticoagulant thromboprophylaxis is recommended for hospitalized IBD patients without severe GI bleeding, especially in moderate to severe cases [386]. Mechanical prophylaxis (e.g., intermittent pneumatic compression) is also recommended in IBD cases with severe bleeding [386]. However, further epidemiological studies are needed to determine the contribution of thromboprophylaxis to physical prognosis and social resources in hospitalized IBD patients. The implementation of thromboprophylaxis in hospitalized IBD patients should be determined considering other risk factors (obesity, steroid use, abdominal surgery, etc.) and the increased risk of bleeding from the GI tract and other organs associated with the intervention [387].

## Postscript

Trends in the diagnosis and treatment of IBD are constantly evolving. With the accumulation of new evidence and the approval of new therapeutic agents, the treatment system for IBD has changed dramatically. In the future, diagnosis using artificial intelligence will be applied to daily clinical practice. Therefore, by the time the next guideline is published, it may be necessary to supplement the guideline with an annual Review and other documents that provide a high level of evidence and new treatments that should be known in clinical practice.

The preparation of these guidelines required a great deal of time and effort, from the process of narrowing down the literature to the preparation of statements and commentaries. This was a truly arduous task. I would like to take this opportunity to express my sincere gratitude to the members of the creation committee and the evaluation committee.
